# Rediscovering Bacteria through Single-Molecule Imaging in Living Cells

**DOI:** 10.1016/j.bpj.2018.03.028

**Published:** 2018-04-18

**Authors:** Achillefs N. Kapanidis, Alessia Lepore, Meriem El Karoui

**Affiliations:** 1 Biological Physics Research Group, Clarendon Laboratory, Department of Physics, University of Oxford, Oxford, United Kingdom; 2 Institute of Cell Biology and SynthSys, School of Biological Sciences, University of Edinburgh, Edinburgh, United Kingdom

## Abstract

Bacteria are microorganisms central to health and disease, serving as important model systems for our understanding of molecular mechanisms and for developing new methodologies and vehicles for biotechnology. In the past few years, our understanding of bacterial cell functions has been enhanced substantially by powerful single-molecule imaging techniques. Using single fluorescent molecules as a means of breaking the optical microscopy limit, we can now reach resolutions of ∼20 nm inside single living cells, a spatial domain previously accessible only by electron microscopy. One can follow a single bacterial protein complex as it performs its functions and directly observe intricate cellular structures as they move and reorganize during the cell cycle. This toolbox enables the use of in vivo quantitative biology by counting molecules, characterizing their intracellular location and mobility, and identifying functionally distinct molecular distributions. Crucially, this can all be achieved while imaging large populations of cells, thus offering detailed views of the heterogeneity in bacterial communities. Here, we examine how this new scientific domain was born and discuss examples of applications to bacterial cellular mechanisms as well as emerging trends and applications.

## Introduction

Single-molecule fluorescence imaging has revolutionized our understanding of the dynamics, heterogeneity, and reaction paths in many fundamental biological mechanisms. Single-molecule methods go beyond ensemble averages and allow us to directly observe the heterogeneity within molecular populations; these methods also track reactions or motions in real-time “movies” that capture the kinetics of individual steps in complicated pathways, often with the added bonus of identifying structural states of the molecular machines or substrates involved (1).

Such measurements, until recently, were confined to in vitro settings and purified components, which offer researchers tight control over conditions to extend the observation span, maximize the spatial and temporal resolution, and permit straightforward addition of interacting molecules. However, such in vitro approaches also come with the caveat of being unable to account for much of the complexity present in cells. For example, the viscous cytosol and its macromolecular crowding may severely affect the rates and equilibria of molecular interactions. One should also consider the presence of fluctuations in biochemical reactions when substrates and enzymes are available at very low copy numbers as well as the effects of the compartmentalization for many processes, the competition between processes for a limiting copy number of multifunctional proteins, and the inability to replicate the complicated cocktail of biomolecules that comprise the natural milieu of living cells.

The desire to preserve the advantages of single-molecule assays while working inside single living cells resulted in the development of the in vivo single-molecule biophysics toolbox (2). The toolbox mostly involves fluorescence-based methods, although innovative force-based approaches have been described. Naturally, this new wave of methods presented a fresh set of challenges for its practitioners; regardless, the approach has already been adopted by many groups and is making an impact by answering long-standing biological questions. In vivo fluorescence detection of single molecules was initially applied to molecular species with low abundance, precisely those for which stochasticity and fluctuations are maximal (2); advances in imaging, many linked to the exciting field of superresolution imaging (3), have extended the approach to essentially any type of cellular protein as well as nucleic acids, metabolites, and membranous structures.

Here, we offer our perspective on studies of single living bacterial cells via single-molecule fluorescence imaging, which is a pillar of the “single-molecule bacteriology” approach that is emerging as a result of technical innovation. Bacteria (such as *Escherichia coli*, *Bacillus subtilis*, and *Caulobacter crescentus*) have been used as model organisms for many decades because they are easy to grow, manipulate, and sequence; the structures of many of their proteins are also available. As a result, bacteria provide fertile grounds for single-molecule method development and subsequent application to mechanistic questions. The methods we discuss are fairly general, and with modifications, they also apply to eukaryotic cells.

This article is not meant to be an exhaustive discussion of all the technical developments and biological applications linked to single-molecule detection in living bacteria. Instead, we discuss crucial developments that led to the birth of the approach and illustrative examples of applications; we also identify emerging trends in the field. For more extensive coverage of the topic, we refer the readers to many excellent reviews (2, 4, 5, 6, 7, 8).

### The route to single-fluorophore sensitivity in single bacterial cells

Understanding of bacterial structure and mechanisms via optical microscopy has been employed as early as the first observations of bacteria by Antonie van Leeuwenhoek in the 17th century. The workhorse for such measurements as well as a model for developing new techniques with ever-increasing spatial and temporal resolution has been the *γ*-proteobacterium *E. coli*, one of the best-studied organisms on the planet. *E. coli*, which lives in the human intestinal tract, has rod-shaped cells that are ∼800 nm in diameter and 2–8 *μ*m in length (depending on their point in the cell cycle), and its cytoplasm is surrounded by a double membrane, with the outer and inner membranes separated by a periplasmic space that contains a ∼10-nm-thick cell wall. *E. coli* cells grow and divide quickly, with a generation time as short as 20 min when nutrients are abundant.

**Figure 1:**
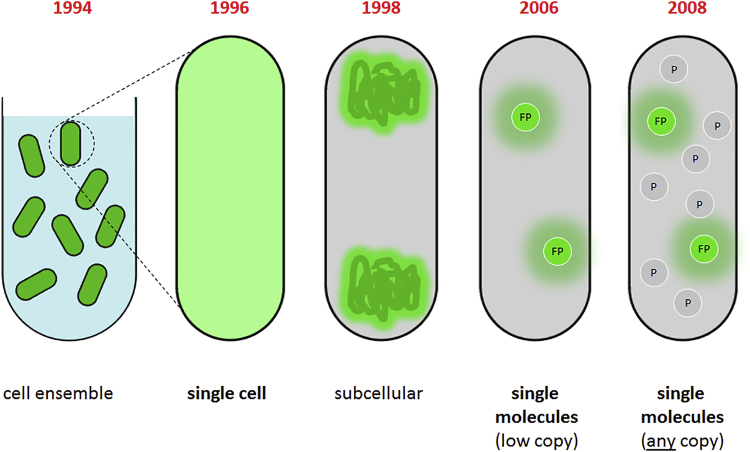
The path to single-molecule detection of proteins inside living bacterial cells. A look at the evolution of imaging bacterial proteins using fluorescent protein fusions is shown. GFP was first developed as a biological probe for gene expression and was used on bacterial populations. Soon thereafter, fluorescence microscopy was focusing on single bacterial cells (10) as well as the subcellular distribution of proteins because there was adequate spatial resolution to do this. In 2006, it became possible to visualize single fluorescent protein fusions (using the Venus-YFP variant (23)) in cells with only a few copies of the protein of interest, and in 2008, the single-molecule detection capability was combined with photoactivation and tracking to study proteins of any copy number inside living bacterial cells (both nonactivated (P) and activated (FP) proteins are represented). To see this figure in color, go online.

A landmark in our ability to dissect mechanisms in *E. coli* came with the advent of green fluorescent protein (GFP) (9), which provided a straightforward, genetic method to tag proteins and, subsequently, many different biomolecules in cells (Fig. 1). The quick transition from studies of GFP-based bacterial populations to single-cell studies led to imaging of subcellular distributions for many bacterial proteins, chromosomal and plasmid DNA, and membrane structures (10, 11).

At that point, there were three main obstacles to achieving single-molecule detection in live cells. The first was limited sensitivity, as the fluorescence light signal emitted by an individual fluorophore is weak, especially considering the cellular autofluorescence background. The second obstacle was limited spatial resolution; the diffraction of visible light limited our ability to resolve objects to within ∼250–300 nm, which was a poor resolution considering the ∼10–20 nm resolution achieved by electron microscopy in fixed samples. The third obstacle was limited photostability; fluorescent proteins tended to stop fluorescing quickly because of irreversible photochemical reactions (“photobleaching”).

It is thus not surprising that the first single-molecule fluorescence studies in living bacteria used multiple copies of a fluorescent probe. An example of such an approach is the detection of messenger RNA (mRNA) molecules in cells. A popular system for this relies on the high-affinity interaction between an RNA hairpin and the MS2 bacteriophage capsid protein; by introducing multiple repeats (typically 24–96) of the hairpin in the mRNA of interest and expressing moderate levels of the MS2 proteins fused to a GFP derivative, one can indirectly tag an mRNA molecule with GFP. This approach has been used for studies of both prokaryotic (12, 13) and eukaryotic cells (14). However, the use of the MS2 system is not without its caveats: the mRNA degradation stability may be altered because of the binding of MS2-GFP proteins, the requirement for many binding sites increases significantly the size of RNA studied, and the nature of signals is indirect, which increases the difficulty of linking signals to phases of transcription or to what is happening for short RNA.

Similar multiprobe approaches have been used for “marking” specific loci on DNA through interactions of DNA-binding proteins with specific DNA-sequence motifs inserted into the bacterial chromosome or plasmids. Two such markers involved use of the fluorescent repressor-operator system (15) (based on transcriptional repressors, such as lacI and tetR) and the parB-*parS* system (based on the binding and subsequent expansion of the plasmid-partitioning protein parB on *parS* sites introduced in the chromosome (16)). The strong fluorescence signal due to the multiple probes allows simple wide-field fluorescence microscopes to be used for detecting the location of specific DNA loci in cells. The use of such markers has led to many advances, such as the analysis of the three-dimensional (3D) organization of the ribosomal RNA operons in the bacterial chromosome (17), the motion of replication forks during DNA replication (18), the mobility and location of bacterial plasmids (19), as well as the direct study of chromosome reorganization and dynamics (20, 21). However, the presence of many DNA-binding proteins on the chromosome may stall polymerase machineries and relocate DNA loci because of energetic destabilization of the DNA conformation in the labeled site; furthermore, the approach does not allow very precise location of the DNA probe, limiting the resolution of the method.

**Figure 2:**
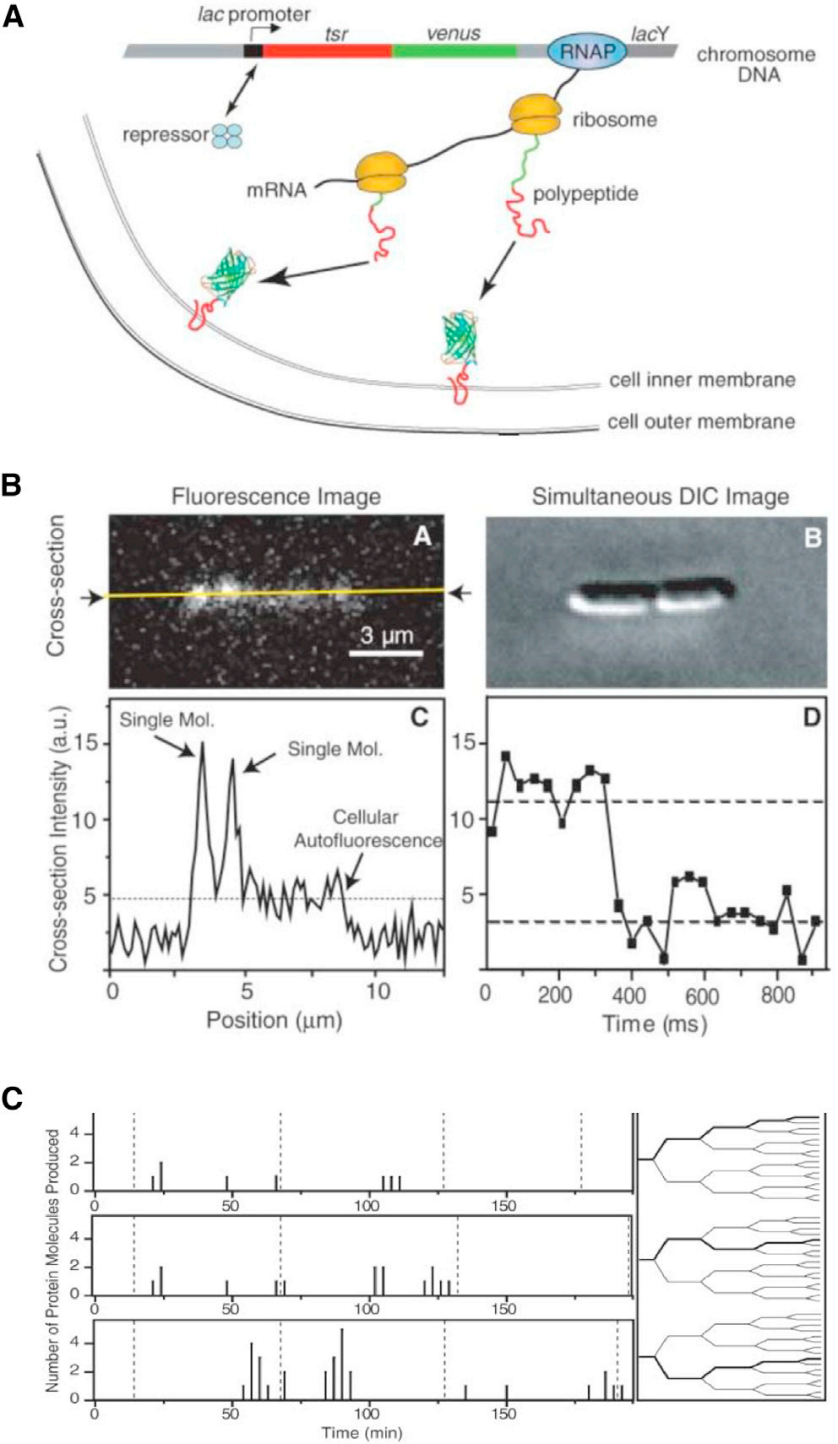
Single-molecule fluorescence detection inside living bacteria. (*A*) A genetic construct occasionally produces a rare protein fusion that localizes on the inner bacterial membrane, which slows down its diffusion and allows detection as a diffraction-limited spot. (*B*) Differential interference contrast and fluorescence images of two bacterial cells show the presence of two fluorescence spots above the autofluorescence background; these spots correspond to single YFP molecules. (*C*) A time-series analysis of protein expression at the single-molecule level is shown. Each protein expression event persists for a significant time, likely because of the rate-limiting steps of fluorescence development in the YFP fluorophore. The figure is adapted from (23). To see this figure in color, go online.

To address these limitations, a concerted effort over the past 15 years has led to a revolution in our ability to detect individual fluorophores in vivo and study bacterial mechanisms at the level of a single protein molecule. Such efforts reflected a general shift toward using quantitative, fluorescence-based cellular imaging to extract accurate molecular concentrations and subcellular localization patterns, as illustrated by concentration measurements in fission yeast (22). The experiments that established the ability to detect single fluorophores in bacteria belonged to a breakthrough study from the Xie laboratory (Harvard University, MA) studying protein expression at the single-molecule level inside *E. coli*. To monitor protein expression in vivo, Yu et al. (23) genetically fused a gene for a fast-maturing version of yellow fluorescent protein (YFP) to a chromosomal copy of a membrane-localization protein fragment (Tsr) (Fig. 2 *A*). The YFP-Tsr fusion was placed under the control of a *lac* operator; stochastic dissociation of the *lac* repressor led to transcription and translation of the YFP-Tsr gene. After folding and membrane insertion, newly synthesized proteins were detected because the protein fluorescence exceeded significantly the cellular autofluorescence and because slow diffusion in the membrane facilitated “detection by localization.” The fluorescence signal persisted for a few frames before the fluorophore bleached in a single step, providing one of the characteristic signatures of a single molecule (Fig. 2 *B*). This method allowed direct counting of the proteins synthesized by a single cell and reported on the timing of their appearance. It was observed that proteins appeared in “bursts” with a large variation in copy number (Fig. 2 *C*), and each burst was attributed to the synthesis of a single RNA molecule. Apart from its biological insight, this study was a methodological breakthrough because it established that one could recover quantitative information about fundamental processes inside a living cell and could directly measure protein cellular location, numbers of protein copies, and their time of appearance.

### First in vivo single-molecule applications: target searches and filament formation

The groundbreaking work of Yu et al. (23) was followed by an impressive study that tracked molecular motion in vivo to address the longstanding question on how transcription factors and other DNA-binding proteins locate their targets in cells (24). Transcription factors such as the *lac* repressor can locate a site on chromosomal DNA fragments with rates up to 100-fold faster than expected on the basis of pure 3D diffusion; theoretical models and experimental in vitro studies had suggested hybrid mechanisms that combine one-dimensional (1D) sliding on DNA with 3D diffusion to account for the experimental observations (25). Elf et al. (24) addressed this question directly using an inventive stroboscopic approach that illuminated a YFP fusion of *lac* repressor for a short time interval during which minimal protein movement occurs because of diffusion. Equipped with this technique and mean-square displacement (MSD) analysis, the group studied both the specific and nonspecific interaction modes of *lac* repressor with DNA and characterized its 3D diffusion in the cytoplasm to show that the repressor spends 90% of time performing 1D sliding on DNA (while dissociating from DNA within 5 ms). These findings supported the combined 1D/3D diffusional mode (“facilitated diffusion”) for target search and paved the way for similar analysis on other DNA-binding proteins.

Subsequent extension using engineering of two proximal *lac* operator sites (26) with different spacing also informed on the “sliding length,” which is the characteristic length-scale over which a protein slides on DNA before dissociating. It was shown that when the interoperator spacing was designed to be 45 bp or shorter, the association rate was significantly lower than that seen with longer spacing, implying that for the long spacing, the operator acted independently; a rate comparison subsequently showed that the sliding length during facilitated diffusion was ∼36 bp. This approach for exploring target search processes in vivo has remained very successful; for example, recent work on the search mechanism and kinetics of the clustered regularly interspaced short palindromic repeats and Cas bacterial endonuclease system (27), aided by powerful microfluidic approaches, has generated a wealth of information on the interactions leveraged by this protein-RNA complex to identify its target with high specificity, albeit at relatively low speed.

**Figure 3:**
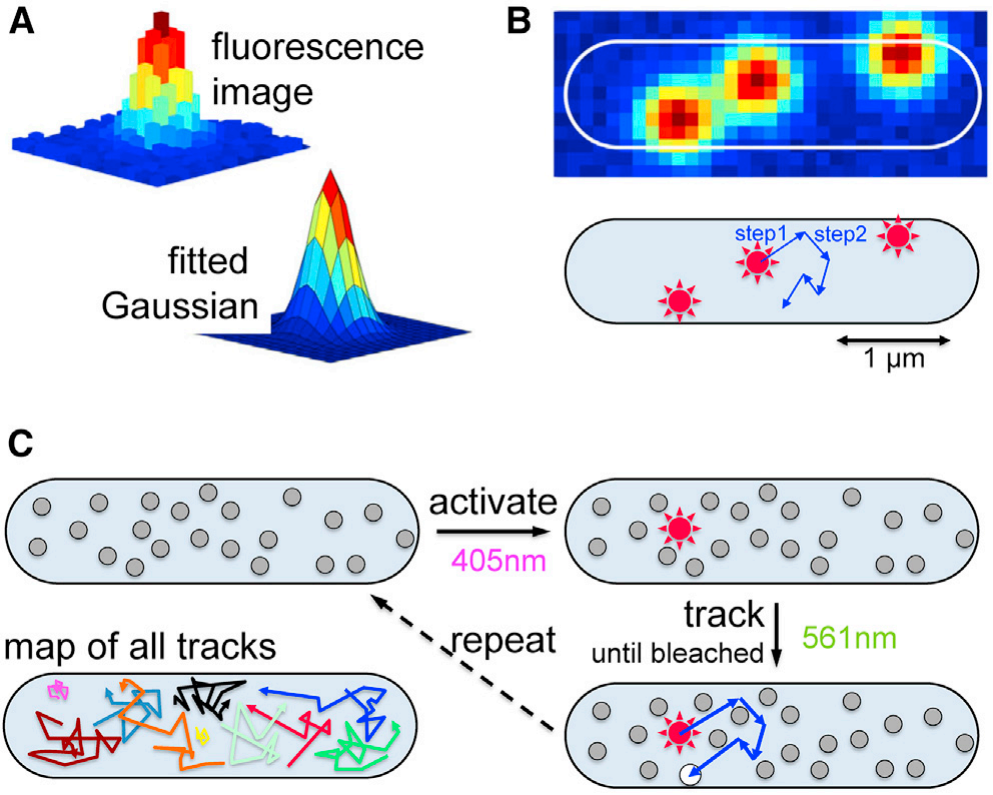
Single-molecule localization and tracking. (*A*) Each single fluorescence molecule is detected as a 2D image with width similar or slightly larger than that of the point spread function (PSF) of the microscope (provided that relatively little motion of the fluorescent molecule occurs during the illumination time during the frame exposure). This image can be fitted with a Gaussian function, and its center can be identified with a precision that depends mainly on the number of photons per molecule per frame. (*B*) Molecules in the cell can be localized, and their motions can be tracked (*lower panel*). Because of the small dimensions of a bacterial cell relative to the size of the PSF (see example of an *E. coli* cell with just three molecules in *top panel*), the presence of many fluorescent molecules leads to a “crowded” situation that does not allow imaging of constantly fluorescent proteins (i.e., autofluorescing without photoactivation) with moderate-to-high copy numbers. (*C*) The principle of photoactivated single-molecule tracking in live bacteria is shown. Proteins are labeled with photoactivatable fusions, which are initially dark and can be turned on stochastically and at very low density using 405 nm light (or ultraviolet light); the activated molecule can be tracked using 561 nm light until it is bleached, and the cycle continues until all molecules are activated, tracked, and bleached, leading to a map of all tracks (*lower left panel*). To see this figure in color, go online.

Significant work, albeit with a different focus, has also been pursued on filaments that are important for cell shape and division in bacteria. Using single-molecule tracking (see Fig. 3, *A* and *B* for the concept) to examine the in vivo diffusion of MreB (a bacterial actin homolog), Kim et al. showed in *C*. *crescentus* the presence of two distinct protein populations: one that diffuses freely (corresponding to single MreB-YFP molecules diffusing in the cytoplasm) and a second that moves more slowly (28). The latter motion was attributed to the addition of labeled MreB molecules to the MreB filament. Indeed, MreB was shown in *B. subtilis* and in *E. coli* to form patch-like filamentous structures approximately perpendicular to the long cell axis, although it did not exhibit treadmilling (29, 30, 31). In contrast, recent work based on imaging of the tubulin homolog FtsZ (the central component of division machinery in bacteria) at high temporal resolution indicated that FtsZ exhibits dynamic treadmilling, which is dependent on its GTPase activity and directs cell wall synthesis at the septum (23, 32).

### Measuring stoichiometries of molecular machines in vivo

An important extension of counting molecules involved studies of the number of protein components that assemble (either stably or transiently) in molecular machinery in cells, i.e., the subunit stoichiometry for a large macromolecular machine. Early work measured the stoichiometry of a stator protein (MotB) and its turnover in the bacterial flagella motor of *E. coli* using stepwise photobleaching (33). Notably, the work was performed in rotating cells, providing an instant functional readout for the motor performance; to achieve this, cells containing a chromosomal copy of MotB-GFP were surface-immobilized via their flagella, and rotation was observed by alternating between bright-field and total internal reflection fluorescence microscopy. To measure the MotB subunit stoichiometry in an active flagella motor, bright fluorescence spots at the center of the cell rotation were examined, and the fluorescence of these spots reflected the superimposition of several signals: the fluorescence from motor MotB-GFP molecules, the fluorescence from nearby MotB-GFP molecules that diffuse on the membrane, membrane-associated autofluorescence, and instrumental background. Because of the continuous exchange of diffusing MotB molecules in the membrane, the membrane-MotB signal bleached much more slowly than the motor-MotB signal; the signals were analyzed as a sum and showed stepwise changes over time, indicative of bleaching of single GFP molecules. Using the fluorescence intensity at the acquisition start provided an estimate of 22 ± 6 for the number of MotB molecules in the motor and ∼200 MotB-diffusing molecules in the membrane.

The same method was used to study the composition and architecture of the bacterial replisome, which is the large multicomponent machine responsible for rapid and accurate DNA duplication in cells (34); most replisome proteins appeared in bimodal distributions that reflected either single replisomes (spaced far apart to be studied as separate point spread functions (PSFs)) or two replisomes spaced more closely than ∼250 nm (and studied together, as they cannot be resolved). This study challenged the long-held notion that the replisome contains only two replicative DNA polymerases because the distributions for two of the polymerase subunits (*ε* and *α*) were centered around ∼3 and ∼6 molecules for the resolved and unresolved replisomes, respectively. These results opened a lively debate about the stoichiometry of protein components at the replisome for many bacterial and eukaryotic systems (35, 36).

### Overcoming “fluorophore crowding” by tracking photoactivated localization microscopy

Despite these impressive advances, three major limitations remained for approaches based on fully fluorescent fusions of proteins: the difficulty in resolving single molecules of proteins with >10 copies per cell, the low photostability of autofluorescent proteins, and the large size of the GFP-like moiety of fluorescent proteins (FPs).

First, proteins with a moderate-to-large copy number were intractable because they would render the typical bacterial cell too “crowded” with fluorescent moieties. Let us consider a bacterial cell with 1 *μ*m in diameter and 4 *μ*m in length. The image width of a single fluorophore reflects the PSF of the microscope (i.e., a Gaussian-like distribution with ∼250 nm width) for a fluorescent molecule that remains immobile within the frame exposure time and is even larger (≫250 nm) for a highly mobile one. Having as few as ∼10 fluorescent molecules in the example cell leads to a crowded situation that prevents single-molecule counting and tracking (Fig. 3, *A* and *B*). It is therefore difficult to study the behavior of most proteins in living bacteria at the native copy numbers. The need for low copy numbers also prevented the collection of large statistics from a single cell, thus limiting the opportunity to study molecular heterogeneities, which may reflect chemical heterogeneity (covalent or noncovalent) or different cellular environments. As a result, to build the needed statistics, data from hundreds of cells were used, obscuring potential differences within a cellular population.

Furthermore, GFPs and their derivatives photobleach very quickly, typically within 100–500 ms at the excitation powers needed for single-molecule detection. However, biological process dynamics occurs over a range of timescales from milliseconds for molecular interactions to tens of minutes (the duration of the cell cycle of a bacterial cell). As a result, processes that occur at longer timescales (e.g., >1 min) were largely inaccessible to direct measurement from the perspective of a single molecule. Finally, the large size of the GFP moiety (a 27 KDa protein of 238 amino acids, forming roughly a cylinder of ∼4 nm in height and ∼2.5 nm in diameter) made the labeling of small proteins and other biomolecules (nucleic acids, reaction substrates, and lipids) difficult or even impossible, either because of loss of biomolecular activity or difficulty with site-specific labeling.

The first step to address these limitations was to control the number of fluorescent emitters in a single bacterial cell via a combination of single-particle tracking with photoactivation, which is a process central to photoactivated localization microscopy (PALM) (37). This method, termed single-particle tracking PALM (spt-PALM) (38), or simply “tracking PALM,” was first applied in studies of protein diffusion on mammalian membranes. In a tracking PALM experiment, proteins are labeled genetically with a photoactivatable or photoconvertible FP, such as mEos2 (39), Dendra2 (40), or PAmCherry (41). As in PALM, single molecules are photoactivated using illumination at a specific wavelength (e.g., 405 nm), imaged for a number of frames upon excitation by a different laser (e.g., 561 nm), and then bleached irreversibly (Fig. 3 *C*). To ensure the presence of very few emitting molecules at any given time, the activation power is adjusted to ensure sparse photoactivation to the point that a cell contains either zero or one molecule at any given frame for the vast majority of the movie frames; this makes linking localizations at consecutive frames much simpler (especially for highly mobile molecules that move significantly during the frame exposure time and between frames). The excitation laser intensity is also adjusted to ensure that each molecular localization relies on enough photons for an acceptable localization precision, that there are enough frames before bleaching (to minimize errors in characterizing diffusion from single-molecule trajectories), and that, combined with the activation-laser power, the fluorophore density in cells remains low.

This “crowd-control” feature of tracking PALM makes it a general method that can address proteins of any copy number in a living cell. Naturally, if one needs to sample all the molecules for a specific protein, the acquisition time will scale with the copy number; imaging all ∼400 molecules of DNA polymerase I in single bacterial cells takes ∼2.5 min, whereas acquisition times for the ∼4000 molecules of RNA polymerase are 10 times longer. However, sampling behaviors and properties (such as the presence and dynamics of molecular clusters) and characterizing diffusion profiles does not require full sampling of the entire set of molecules in each cell.

After recording the videos, molecules are localized in each frame by fitting each molecular image to a two-dimensional (2D) Gaussian, as in the PALM experiments; localizations between frames (occurring within a certain radius that reflects limits based on the diffusion for a protein of a given size) are linked to generate trajectories, which are analyzed to characterize diffusion per molecule in single cells. Because the number of sufficiently long tracks can be large, dense maps of diffusion can be generated per cell and can probe cellular microenvironments and molecular subpopulations. The spatial detail offered by this approach is much finer than that offered by diffraction-limited microscopy because the resolution is now dictated by the localization precision and not by the PSF width.

**Figure 4:**
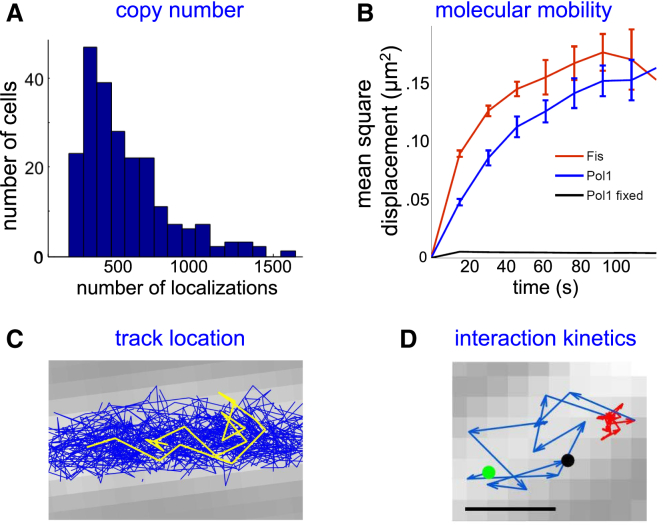
Main single-molecule fluorescence observables inside living bacteria using fluorescent proteins. The observables apply for both autofluorescent and photoactivatable proteins, although the latter will provide higher statistics for counting and tracking. (*A*) The number of localizations per cell is shown, corresponding loosely to the protein copy number per cell. (*B*) Molecular mobility can be examined using plots of mean-square displacement (MSD) (*pictured*) or the cumulative distribution function (which plots the cumulative probability of finding a molecule within a certain distance after a certain time); this information can also be converted into apparent diffusion coefficients per track. In the example, a DNA polymerase (Pol1) in fixed cells shows no significant motion, whereas in live cells, it shows significant displacements until the confinement effects cause saturation of the MSDs; a smaller DNA-binding protein, Fis, shows faster motion. (*C*) Track location can be examined relative to the cell boundaries, relative to all other tracks (as pictured), relative to tracks with the same or different mobility, and relative to cell landmarks monitored in a different detection channel. (*D*) Time-series analysis of individual tracks can provide information about interaction (binding) kinetics identified by changes in the molecular mobility. In the example, a DNA polymerase molecule identifies its target, performs DNA synthesis, and resume its target search. Each step is 15 ms. Scale bars, 500 nm. The example figures are taken from (50). To see this figure in color, go online.

Tracking PALM offers a wealth of observables and information. First, it offers the number of localizations and tracks as an estimate of the copy number of the protein of interest (Fig. 4 *A*); this relies on the fact that the results (ideally) reflect cycles of a molecule being photoactivated, providing either a single localization or a short track, and then bleaching.

Second, the protein-diffusion landscape (ranging from immobile molecules to freely diffusing GFP, which has a diffusion coefficient of ∼7 *μ*m^2^/s (42)) can be recovered by analyzing the displacements within tracks to calculate apparent diffusion coefficients per track (considering 2D diffusion; Fig. 4 *B*). For proteins of moderate size (>30 KDa), the intracellular motion in the crowded intracellular environment tends to be slow enough to be able to capture positions accurately from images of single molecules (thus avoiding complications due to confinement and image blurring). This in turn can help sort tracks into groups of different mobility and examine how this distribution changes as a function of cellular physiological states.

Third, there is substantial spatial information (Fig. 4 *C*) deduced from the relative locations of the tracks of protein molecules belonging to different mobility groups, e.g., one can identify where immobile molecules localize versus ones that diffuse rapidly. Further spatial information can be obtained by analyzing track location in relation to cell landmarks and borders; the latter can be obtained from bright-field microscopy and diffraction-limited fluorescence (e.g., membrane location, sites on the chromosome, and large intracellular clusters).

Fourth, the switch between different modes of protein mobility can inform reaction or binding kinetics (Fig. 4 *D*). This may be obvious because of the site of the localization or clear diffusion changes (e.g., very mobile → immobile → very mobile); statistical approaches (based on hidden Markov modeling) have also been described to discern more complex kinetics of interconversion between diffusive states (43).

All these capabilities are not without caveats. For example, counting molecules accurately is complicated by sources of undercounting and overcounting, requiring knowledge of the detection efficiency of a specific FP in a certain cellular context. Blinking of photoactivatable FPs leads to overcounting; a photoactivated molecule can enter a dark state reversibly until it eventually bleaches. To account for blinking, a threshold time can be used to join consecutive appearances of a single molecule. A clever way to deal with blinking is to characterize the photoactivation, blinking, and bleaching kinetics for the fluorophore employed (44) and use the results to correct the molecule count. In contrast, incomplete FP folding and maturation may lead to undercounting, especially because the maturation time t_0.5_ (time to activate 50% of all labeled proteins) of even the fastest-maturing photoactivatable FPs is in the 20–60 min timescale (41, 45), which is a relatively slow timescale considering that rapidly growing bacterial cells can divide within ∼20 min; approaches that measure the dark fraction of photoactivatable FPs are becoming available.

Other complications include the presence of complex diffusion modes that feature interconversions between states, the difficulty in capturing very fast species, the tendency of many auto-FPs to oligomerize (distorting interactions to the point that the localization and clustering status of proteins is altered (45, 46)), and the technical complexity of two-color measurements (to check colocalization) in the same single cell.

### First applications of tracking PALM in bacteria

The first tracking-PALM application in bacteria involved an elegant study of the intracellular diffusion of the bacterial tubulin analog, FtsZ (47); the study relied on a FtsZ-Dendra fusion. Using single-frame displacements, FtsZ molecules were sorted into immobile (with displacements reflecting just localization error) and mobile ones (with an average displacement of ∼200 nm). Most immobile molecules localized close to mid-cell, which was consistent with them being part of the Z-ring. Taking advantage of the massive set of tracks, the authors selected long tracks to examine the mode of diffusion further, showing the diffusing molecules forming an apparent helical filamentous structure and displaying anomalous diffusion, either because of their continuous assembly-disassembly in helical FtsZ filaments or their interaction with another helical membrane-associated structure.

This FtsZ study, as with earlier diffusion studies of low-copy-number proteins (24), focused on proteins that diffuse slowly either because of their association with the membrane or stable cellular structures (e.g., DNA and cytoskeleton). English et al. (48) moved tracking PALM into the domain of fast-diffusing cytoplasmic proteins; to achieve this, the study employed stroboscopic illumination (24), wherein very short illumination of a diffusing molecule minimizes its displacement (and thus, motion-blurring) within a frame, facilitating the localization of molecules (the images of which become near-diffraction-limited) and an accurate MSD analysis. To show that the new method could capture fast diffusion in vivo, English et al. characterized the diffusion of mEos2. Using an illumination time of 1 ms (during which little motion occurs because the GFP diffusion coefficient in the bacterial cytoplasm is ∼7 *μ*m^2^/s; see (42)) and a frame exposure time of 4 ms, they achieved efficient mEos2 tracking and characterized its diffusion and subcellular distribution. The fast mEos2 motion led to deviations from the linear relation between MSD and diffusion time due to cellular confinement; however, simulations that account for projection and confinement effects showed that mEos2 diffuses freely and explores the entire cell without being perturbed by large structures such as the nucleoid. Similar analysis on the ribosomes showed much slower diffusion as well as anomalous diffusion, the latter being attributed to the interaction of ribosomes with the chromosome in the nucleoid (49).

**Figure 5:**
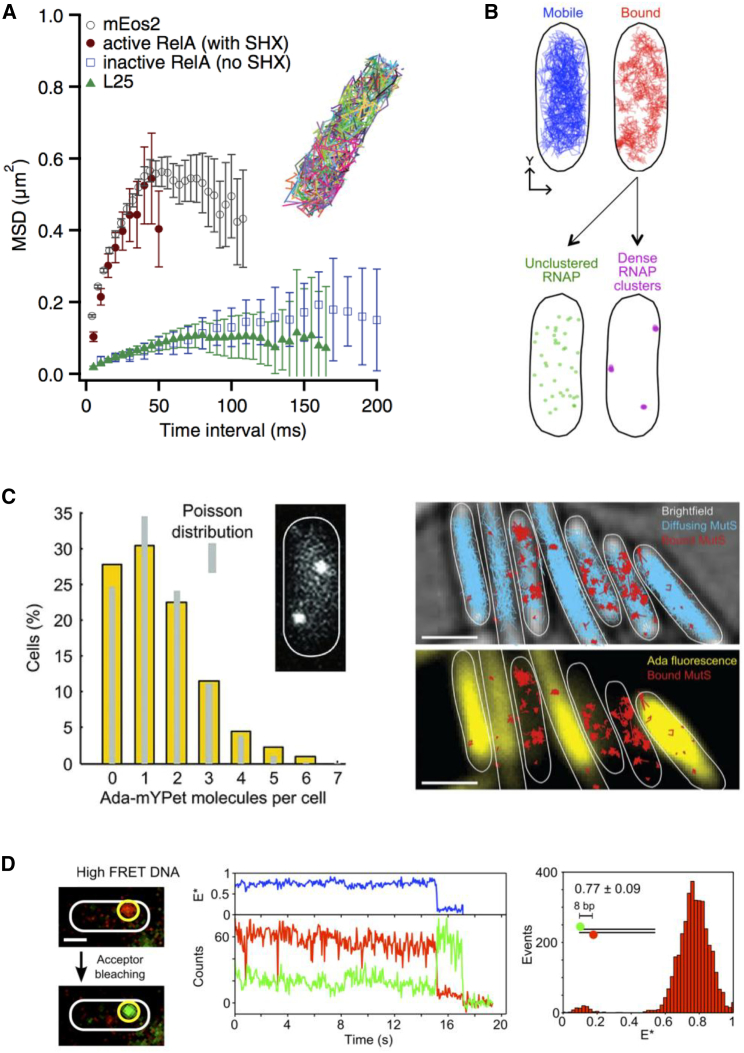
Applications of single-molecule imaging in living cells. (*A*) Using diffusion standards, it was shown that RelA diffusion, when inactive, is slow, matching that of ribosomes. The figure is adapted from (48). (*B*) The spatial profile of RNA polymerase shows that actively transcribed genes tends to be present in the nucleoid periphery. The figure is adapted from (51). (*C*) Single-molecule imaging of transcription factor Ada shows that some cells do not contain any molecule of this factor (*left*), delaying DNA-damage responses. This heterogeneity is also reflected in the diffusion profile of the MutS protein (*right*), which recognizes DNA mismatches that form when Ada is not present to repair damaged DNA molecules. There are many more DNA-bound MutS molecules (reflecting the presence of mismatches) in cells with low Ada content compared to cells in which Ada is abundant. The figure is adapted from (55). (*D*) Electroporated DNA can provide measurements of single-molecule FRET and distances within living cells. The figure is reproduced with permission from (69). To see this figure in color, go online.

English et al. also examined the mobility of RelA, a protein that, during the stringent response (a bacterial adaptation to starvation stress), produces pppGpp, a small molecule that acts as an alarm signal that changes gene expression by redirecting transcription by RNA polymerase. RelA was largely immobile under nonstarvation conditions (during which RelA is inactive) and matched the diffusion profile of ribosome, likely because inactive RelA binds to the ribosome (Fig. 5 *A*). In contrast, under starvation, RelA motion was much faster and similar to free mEos2; these results establish that RelA leaves the ribosome while active, supporting a hopping model for its function. The results also suggested that hopping happens more slowly than initially thought because the results were inconsistent with immediate RelA rebinding to another ribosome upon its dissociation.

Further sensitivity and resolution improvements allowed continuous (i.e., without stroboscopy) protein tracking in the cytoplasm, as in a DNA-repair study that examined the diffusion of DNA polymerase I (Pol1) and ligase, which are proteins that perform DNA synthesis and ligation in DNA repair and replication (50). Using chromosomal PAmCherry fusions, this tracking-PALM approach recovered copy numbers in close agreement with the Pol1 and Lig copy numbers in the literature (∼400 and ∼200 copies, respectively), supporting its quantitative nature. Tracking analysis showed that the proteins diffused rapidly in undamaged cells. In contrast, cell exposure to a DNA-damaging agent called methyl methanesulfonate (MMS) generated a fraction of bound Pol1 and ligase, with the binding sites being distributed randomly in the nucleoid; control experiments showed that the transient binding was specific to DNA damage. Pol1 and ligase tracks also acted as nucleoid “highlighters” in single cells, likely because of their strong association with the nucleoid via nonspecific interactions. Furthermore, the availability of many thousands of tracks allowed the selection of long tracks that displayed entire repair cycles, including target searching for a lesion, chromosome binding for repair DNA synthesis, and resumption of diffusion (Fig. 4 *D*). These ways to extend the fluorophore survival and PSF-width measurements helped to make one of the first direct measurements of in vivo reaction times by single proteins, which amounted to repair times of 2.1 s for Pol1 and 2.5 s for the ligase. The number of tracks and the bound fraction for these two proteins also provided a direct report of repair rates and search times at the single-cell level for many MMS concentrations and incubation times. Repair rates increased within minutes of MMS exposure and led to saturation of DNA repair intermediates, with the overall repair activity being limited by upstream pathway steps. The study was an elegant demonstration of how tracking PALM can provide systems-wide views of crucial molecular processes in single cells.

### Examples of tracking PALM applications to transcription, translation, and DNA repair

The previous reports established the imaging methods, data analysis, and main observables offered by tracking PALM. Soon after these developments, applications to crucial biological questions and discoveries based on unexpected observations started to emerge. One such application is related to the spatial organization of RNA polymerase (RNAP), the main engine of transcription. Stracy et al. (51) used tracking PALM to differentiate between the pool of diffusing RNAPs and the pool of RNAPs bound to DNA, either on promoters or transcribed genes; each pool comprised ∼50% of all RNAPs in cells grown in minimal media. The study used comparisons between the RNAP diffusion landscape in *E. coli* strains with different DNA content to show that mobile RNAPs explore the whole nucleoid while searching for promoters, spending ∼85% of their time in nonspecific interactions with DNA. On the other hand, systematic analysis of RNAP localizations revealed that unclustered bound RNAPs showed low levels of transcription throughout the nucleoid; however, dense clusters of transcribing RNAPs formed predominantly at the nucleoid periphery. The study combined tracking PALM and structured illumination microscopy to show that during faster growth, the clustering of transcribing RNAPs increases, leading to a dramatic phase separation between the densest RNAP clusters and the densest nucleoid regions (Fig. 5 *B*). This study benefited from an earlier PALM study of RNAP in fixed bacterial cells (52); although the RNAP and chromosome dynamics were lost after fixation, the fixed-cell work set the stage for live-cell study and captured spatial features (e.g., the presence of different cluster sizes under different growth conditions) that were confirmed and extended in live cells. In this respect, studies of fixed cells can serve as excellent entry points for single-molecule imaging in live cells for many systems. Furthermore, correlative studies on the same cells, imaged both live and fixed, are possible, as has been shown in a study that combined PALM of RNAP in live cells, followed by fixation and superresolution imaging of chromosomal DNA in fixed cells (53).

Another recent tracking-PALM study leading to intriguing discoveries focused on the nucleotide excision repair pathway in *E. coli* (54); this mechanism protects genomes against mutagenic DNA damage by removing damaged nucleotides and filling in the resulting gap with intact DNA. In *E. coli*, the lesions are recognized by UvrAB, a complex of UvrA and UvrB proteins. Analysis of UvrA diffusive behavior identified a population of immobile DNA-bound molecules and a population of slowly diffusing molecules, which probably resulted from transient interactions with DNA. The proportion of DNA-bound molecules and the duration of binding increased markedly in the presence of DNA damage. In contrast, UvrB was shown to be mostly not localized to DNA in undamaged cells, but was recruited by UvrA to the lesions. This led to a new model for the first steps of nucleotide excision repair, in which the search for lesions is performed by UvrA alone (and not the UvrAB complex, as previously thought), which then recruits UvrB in an ATP-dependent manner.

A similar approach was used to identify the presence of DNA mismatches in cells by tracking the mismatch recognition protein, MutS (55). When cells were exposed to a DNA-damaging agent, a high proportion of MutS (56%) appeared bound to DNA, indicating that MutS had detected a mismatch; importantly, increased MutS binding occurred only in cells in which the Ada repair protein was expressed at a low level or was completely absent, which precluded the repair of DNA lesions and led to high levels of mismatch (Fig. 5 *C*). This study thus demonstrated for the first time how stochastic fluctuations in the expression of a DNA repair protein can lead to mismatches and genetic modifications.

Tracking PALM has also proven extremely useful for the study of translation, as was also shown in some of the first studies using this method (48, 49). As the proteins of the translation machinery are usually very abundant, ensuring the photoactivation of only a very small subset is essential for such studies. A fusion of the ribosomal S2 protein to mEos was used to show that most ribosomes diffuse slowly (with an observed diffusion coefficient D ∼0.04 *μ*m^2^/s), whereas some molecules showed faster diffusion (56). Treatment with an antibiotic that stops transcription led to increased diffusion, which was consistent with partial disassembly of the ribosomes.

An interesting recent development is the use of split mEOS for bimolecular fluorescent complementation coupled with PALM, which permits the detection of the dynamics of complex formation. This has led to the characterization of the interaction between MreB and EF-Tu, one of the bacterial translation elongation factors (57).

### Using organic fluorophores to minimize bleaching and increase photon counts

Although FPs have opened the possibility of single-molecule observations in cells, they have several disadvantages, including their relatively low brightness and photostability as well as their tendency to promote protein oligomerization (46). One way to overcome bleaching is to adjust the illumination conditions to detect slow molecular processes despite the limited “photon budget” of FPs; e.g., one can use different combinations of excitation intensity and exposure times to detect molecules that remain immobile during the frame exposure time. This technique can identify the fraction of DNA-binding proteins bound to DNA from the fraction that is freely diffusing and can report on target-search processes (24).

However, a more general approach to address fast bleaching and other caveats of FPs is to switch the intracellular labels to organic fluorophores, which are much smaller, brighter, and more photostable than FPs (58). One way to achieve this is via the use of self-labeling protein tags, such as the Halo-Tag (59) or the SNAP tag (60), in living cells. Specificity is achieved by constructing a fusion of the protein of interest to the protein tag, which is then covalently bound by the organic fluorophore. Such self-labeling tags have been widely used in eukaryotic cells for visualization in different cellular and subcellular compartments (61, 62, 63, 64). In bacteria, the use of the self-labeling tags is potentially more limited because of the low permeability of the bacterial cell envelope to the fluorophores. However, the possibility of labeling proteins in both the periplasmic and cytoplasmic compartments of *E. coli* cells using the Halo-tag tetramethylrhodamine ligand has been recently established (65). Fully functional Halo-tag fusions in both the periplasmic and cytoplasmic compartments were imaged and compared with the corresponding GFP construct, showing the same specificity in the labeling between the Halo-tag and the GFP proteins (65). Moreover, Halo-tag-based and SNAP-tag-based protein fusions have been used for superresolution imaging of type I and III secretion systems in *Salmonella enterica* cells (66). The combination of SNAP- and Halo-tag labeling also allowed for dual-color labeling, indicating that this technology can be used to follow the assembly of multiprotein complexes.

The use of self-labeling tags has many advantages over FPs; several fluorophores with multiple colors are available, and more are being developed (62). It has also recently been shown that self-labeling tags are a very attractive choice for analysis of low-copy-number proteins because they can count very low-abundance proteins even on a simple epifluorescence microscope with labeling efficiency at least as good as with FP-based fusions while maintaining single-molecule sensitivity (67). Moreover, the development of photoactivatable organic fluorophores (68) will greatly advance the use of self-labeling tags for single-molecule tracking and superresolution studies. As with any technique, self-labeling tags do have limitations; of particular importance is the need for careful washing steps after labeling to eliminate nonspecific signals that can limit the time resolution at which molecular processes can be analyzed. Furthermore, the ideal labeling concentrations may need to be adjusted for each fluorophore and each bacterial species because of the different membrane permeability properties or the presence of efflux pumps that reduce the intracellular fluorophore concentration. Nevertheless, this technology presents an attractive complementary solution to the use of FPs.

Another approach to enable the use of organic fluorophores inside live bacteria relies on delivering in vitro labeled biomolecules into cells by electroporation. Electroporation, which relies on cell exposure to short but intense electrical pulses, leads to the formation of transient pores in the bacterial membrane, thus allowing internalization of labeled biomolecules placed in the electroporation cuvette; cell washing before imaging removes any noninternalized molecules. Millions of cells can be electroporated simultaneously, and the efficiency of loading is tunable because of its dependence on the concentration of the electroporated molecule in the cuvette and the applied voltage. Electroporation works well for internalization of DNA, RNA, and proteins into both bacteria and yeast (69) and is particularly amenable to internalization of small-to-moderate-size proteins (70). Because organic fluorophores are small, one can site-specifically introduce two probes per protein or DNA to allow single-molecule Förster resonance energy transfer (smFRET) measurements; this capability allows measurement of intramolecular distances in vivo and can recover conformational states in native environments, which is a clear advantage over self-labeling tags that still rely on protein domains (Halo/SNAP domains) with sizes similar to FPs.

An example of the use of electroporated substrates involved the delivery of Cy5-labeled transport RNA (tRNA) molecules in *E. coli* (71). Single-particle tracking of single internalized tRNA molecules over several seconds led to the observation of two diffusive species in cells: a fast one with a diffusion coefficient of ∼8 *μ*m^2^/s that was consistent with free tRNA and a slow one (D ∼0.1 *μ*m^2^/s) that was consistent with tRNA bound to larger complexes; these results suggested that a large fraction of internalized fluorescent tRNA (>70%) appear to diffuse freely in the bacterial cell. Further work has been done using DNA substrates recognized by endogenous proteins. One such example used electroporation of gapped DNA and in vivo smFRET to show that the gapped substrate is severely bent in vivo, as was also found using structural smFRET in vitro (72).

One should consider, however, that electroporation is a fairly drastic approach because its application can affect growth in many treated cells and requires time for recovery; electroporation also requires in vitro protein labeling, which can be complicated for some proteins. Furthermore, the delivery is not done at the copy number level of native proteins; rather, the delivered protein acts as a representative of the internal pool.

### What about the future?

Single-molecule imaging in living bacteria has already reported extensively on many properties of proteins and other biomolecules, including copy number, subcellular distribution, mobility, and interactions with other molecules and cellular structures; the insights from this information have often transformed our mechanistic understanding. It is also clear that there are many possible extensions of the approach that will enhance its capabilities, applicability, and appeal among bacteriologists. Such extensions include improved fluorophores and labeling methods; advanced microscopies for higher resolution, higher content, and lower photo damage; force sensors; correlative approaches; advanced microfluidics and data analysis routines; mathematical modeling; and extension to other bacteria and applications.

Despite the introduction of methods that rely on organic fluorophores or make prudent use of the limited photon budget, bleaching is still a serious limitation, especially if the minute-to-hour timescale is to be explored. Nonbleaching probes with high photon counts would be ideal for long-term observations that record extended molecular “histories”; fluorescent quantum dots (73) and nanodiamonds with nitrogen-vacancy centers (74) hold promise for this direction. However, because these probes are bulky, internalization and labeling remain a challenge. Smaller versions of these probes exist, but their photophysics is complex; furthermore, the photon counts for nanodiamonds are substantially lower than those for organic fluorophores.

Ways to site-specifically label molecules and sites of interest with individual fluorophores are also seriously limited. Labeling most proteins with FPs is relatively straightforward but can result in nonfunctional or partially functional fusions; an exciting development is the possibility of labeling proteins in vivo with unnatural fluorescent amino acids (e.g., using coumarin-containing amino acids) using orthogonal translation systems. This strategy, which is still very challenging (75) and requires a recoded genome to reassign codons, is likely to bear fruit in the near future. Besides proteins, labeling other molecules such as single specific sites on the chromosome, mRNA molecules, or polysaccharides is still very complicated, if at all possible. Use of specialized biological machinery (e.g., the CRISPR/Cas systems or site-specific DNA-modifying enzymes) may help significantly in these labeling efforts.

An attractive option is to maximize the information content per photon, which can in turn increase the spatiotemporal resolution or observation span. A new superresolution method called MINFLUX, which combines the illumination pattern of stimulated emission depletion with the photoactivation principle used in localization microscopies such as PALM, is already transforming our ability to localize molecules with high precision (down to 1 nm) and track machineries, such as the 30S ribosome subunit, with 100-fold better temporal resolution in bacteria (76).

Our ability to see conformational states and changes in living cells will benefit from streamlining the smFRET measurements relying on electroporated molecules. More photostable fluorophores, use of unnatural amino acids for labeling by cell-permeable reactive conjugates, and ways to control the density of fluorescence resonance energy transfer (FRET) pairs in vivo (extending the concept of switchable FRET (77)) may make in vivo smFRET as useful a tool as its in vitro counterpart.

There is also ample room to explore in vivo force generation. Despite the routine application and measurement of piconewton forces in in vitro single-molecule measurements based on optical tweezers, magnetic tweezers, or atomic-force microscopy, such measurements cannot currently be performed inside living cells. Transferring approaches that rely on genetically encoded force sensors or internalized versions of chemically synthesized force sensors from eukaryotic systems (78) to bacteria should allow us to get the first glimpses of such measurements in bacterial cells, opening new domains.

Comparison of fluorescence imaging information with other microscopies (e.g., electron microscopy, which can provide the overall cellular context, whereas fluorescence gives dynamics and structure for specific components) and genome-wide approaches (based on single-cell next-generation sequencing) will leverage the content of spatial information and inform on transient but biologically important 3D interactions between different parts of the bacterial chromosome.

The existing imaging efforts are already considered “big-data” projects, providing large data sets that require sophisticated image- and time-series analyses to probe protein location and mobility. New algorithms for data analysis coupled with automation and robust microfluidic platforms that allow reproducible and unattended operation will reduce the tedium and increase the reproducibility and throughput of the measurements. The microfluidic platforms can be used to control the size/shape of bacterial cells, to introduce cells to different microenvironments that test responses to environmental exposure, or to monitor interactions (cooperative or adversarial) between different bacterial species. There are already many excellent examples of such integrated efforts (79), and we expect this trend to continue.

The availability of large data sets and the quantitative, statistically robust, imaging-based information on molecular properties in vivo also provides excellent input for the construction of mathematical models that describe many processes, from molecular diffusion in vivo to the function of intricate gene networks and molecular machines (80). The improvement in resolution and throughput will further expand this trend, which will also be helped by the emergence of engineered synthetic cells produced by synthetic biologists.

Finally, the current methods have so far been applied only to a small number of organisms out of the vast universe of available bacteria. The streamlining of the instrumentation and analysis and the diffusion of knowledge to the bacteriology community should fuel an expansion of the single-molecule approach to many more bacteria, environments, and complex systems, such as clinical samples, bacterial biofilms, and environmental bacterial communities. These efforts should continue to reveal many well-hidden secrets of these tiny but continuously fascinating creatures.

## Author Contributions

A.N.K., A.L., and M.E.K. wrote the manuscript. A.N.K. prepared the figures.
